# Prognostic value of 17-Gene genomic prostate score in patients with clinically localized prostate cancer: a meta-analysis

**DOI:** 10.1186/s12885-024-12389-1

**Published:** 2024-05-23

**Authors:** Feilun Cui, Xuan Tang, Changfeng Man, Yu Fan

**Affiliations:** 1 Department of Urology, Affiliated Taizhou Second People’s Hospital of Yangzhou University, Taizhou, 225500 China; 2 Department of Urology, The Fourth Affiliated Hospital of Jiangsu University, Zhenjiang, 212001 China; 3 Cancer Institute, The Affiliated People’s Hospital, Jiangsu University, No. 8 Dianli Road, Zhenjiang, Zhenjiang 212002 China

**Keywords:** Genomic prostate score, Prostate cancer, Distant metastases, Biochemical recurrence, Prostate cancer–specific mortality, Meta-analysis

## Abstract

**Background:**

The 17-gene Genomic Prostate Score (GPS) test has been clinically employed to predict adverse prognosis in prostate cancer. In this meta-analysis, we aimed to evaluate the prognostic value of the 17-gene GPS in patients with prostate cancer.

**Methods:**

Potentially relevant studies were obtained by searching PubMed, Web of Science, Embase databases from their inception to December 1, 2023. Studies were considered eligible if they evaluated the association of the 17-gene GPS with distant metastases, biochemical recurrence, or prostate cancer–specific mortality (PCSM) in prostate cancer patients. To estimate the prognostic value, we pooled the adjusted hazard ratio (HR) with 95% confidence intervals (CI) for the high versus low GPS group or per 20-unit increase in GPS.

**Results:**

Seven cohort studies that reported on 8 articles comprising 1,962 patients satisfied the eligibility criteria. Meta-analysis showed that per 20-unit increase in GPS was significantly associated with distant metastases (HR 2.99; 95% CI 1.97–4.53), biochemical recurrence (HR 2.18; 95% CI 1.64–2.89), and PCSM (HR 3.14; 95% CI 1.86–5.30). Moreover, patients with high GPS (> 40 points) had an increased risk of distant metastases (HR 5.22; 95% CI 3.72–7.31), biochemical recurrence (HR 4.41; 95% CI 2.29–8.49), and PCSM (HR 3.81; 95% CI 1.74–8.33) than those with low GPS (≤ 40 points).

**Conclusions:**

A higher 17-gene GPS significantly predicts distant metastases, biochemical recurrence, and PCSM in men with clinically localized prostate cancer. However, large-scale multicenter prospective studies are necessary to further validate these findings.

**Supplementary Information:**

The online version contains supplementary material available at 10.1186/s12885-024-12389-1.

## Background

Prostate cancer is the fifth most common malignancy worldwide, accounting for approximately 20% of all new cancer cases in men [1]. In 2023, the National Cancer Institute estimated about 288,300 new cases of prostate cancer in the United States and 34,700 deaths from this disease [2].

Despite early screening for prostate cancer using prostate-specific antigen (PSA), patients with metastatic disease still had a poor 5-year survival rate [3]. Nevertheless, around 20–30% of men with prostate cancer will experience recurrence within five years after the initial treatment [4]. Traditionally, the risk stratification for prostate cancer is based on a combination of PSA level, Gleason score, and clinical stage of the tumor at diagnosis [5]. However, these prognostic factors are not sufficient to accurately predict the recurrence and survival. Therefore, there is an urgent need to explore additional specific prognostic indicators.

The incorporation of tissue-based genomic biomarkers into prostate cancer patients can provide valuable prognostic information [6]. One such biomarker is the 17-gene Oncotype DX Genomic Prostate Score (GPS), which has been introduced to predict the aggressiveness of prostate cancer [7]. This assay quantitatively detects the expression of 17 genes in messenger RNA obtained from prostate biopsies using a reverse transcriptase polymerase chain reaction. In addition to aggressiveness of the tumor, the GPS also provided valuable prognostic information of prostate cancer. A few studies [8–13] have utilized the GPS to predict adverse outcomes in prostate cancer patients, such as distant metastasis, biochemical failure, biochemical recurrence, and prostate cancer-specific mortality (PCSM). However, these findings were limited by small sample sizes of prostate cancer patients.

The prognostic value of the GPS has not been extensively summarized in previous systemic review and meta-analysis. To fill this knowledge gap, we conducted this meta-analysis to evaluate the prognostic value of the 17-gene GPS in patients with prostate cancer.

## Materials and methods

### Study guideline and ethics approval

This study was reported based on the guideline of the Preferred Reporting Items for Systematic Reviews and Meta-Analyses [14]. Ethical approval was not required because the study did not involve individual patient data.

### Search strategy

Two authors independently searched PubMed, Web of Science, Embase databases from their inception to December 1, 2023. The following search keywords were used in combinations (Supplemental Text S1): (“17-Gene Genomic Prostate Score” OR “Oncotype DX Prostate Cancer Assay”) AND (“biochemical recurrence” OR “biochemical failure” OR “metastasis” OR “mortality” OR “death” OR “survival”). We also scanned the reference lists from included studies and pertinent reviews to identify additional studies. No language restriction was imputed for literature search.

### Study selection

Studies were included if they met all the following criteria: (1) type of study: prospective or retrospective cohort as design; (2) population: patients diagnosed with prostate cancer; (3) predictor: elevated GPS level; (4) comparison: high versus low GPS or per 20-unit increase in GPS; (5) outcome measures: distant metastases, biochemical recurrence, or prostate cancer–specific mortality (PCSM); and (6) reported multivariable adjusted risk estimates of abovementioned outcomes associated with GPS. Articles published in meeting abstract, review, letters, or comment were not included. Two independent authors performed the study selection process. Disagreements were settled through consensus between the two authors or consultation with the corresponding author. For multiple articles using the same population, we selected the longest follow-up article.

### Data extraction and quality assessment

Two independent authors performed data extraction and quality assessment. The authors settled their disagreements through discussion with the corresponding author until they reached a consensus. For each included study, we extracted the first author’s name, publication year, country of origin, study design, patient number, age at diagnosis, treatment approach, definition of biochemical recurrence, cutoff value of GPS, length of follow-up, outcome measures, hazard ratios (HR) with 95% confidence intervals (CI) in fully adjusted analysis, adjusted covariates, and domains for assessing methodological quality. The study quality was evaluated in accordance with a-9 points Newcastle-Ottawa Scale (NOS) [15]. A study with score ≥ 7 was considered as to have high quality.

### Statistical analysis

All meta-analyses were run using STATA 12.0 (Stata Corporation, Texas, USA). We pooled the fully adjusted HR with 95% CI to calculate the prognostic value of GPS. Cochrane Q test and *I*^2^ statistic were used to determine the heterogeneity between studies. The presence of significant heterogeneity was defined by a *p-*value < 0.10 for Cochrane Q test and an *I*^2^ statistic > 50%. In the case of significant heterogeneity, we chose a random-effects model to pool the data. Otherwise, a fixed-effects model was used for the analysis. However, we only chose a random-effect model for all the analyses due to the obvious heterogeneity in patients’ characteristics. Subgroup analyses were performed according to the study design, sample size, treatment options and follow-up duration. Begg’s test [16] and Egger’s test [17] were conducted to assess the publication bias when more than five studies reported the same outcome. To observe the credibility of the pooling results, we performed the sensitivity analysis by excluding individual study at each turn to recalculate the pooling risk summary.

## Results

### Search results and study characteristics

**Fig. 1:**
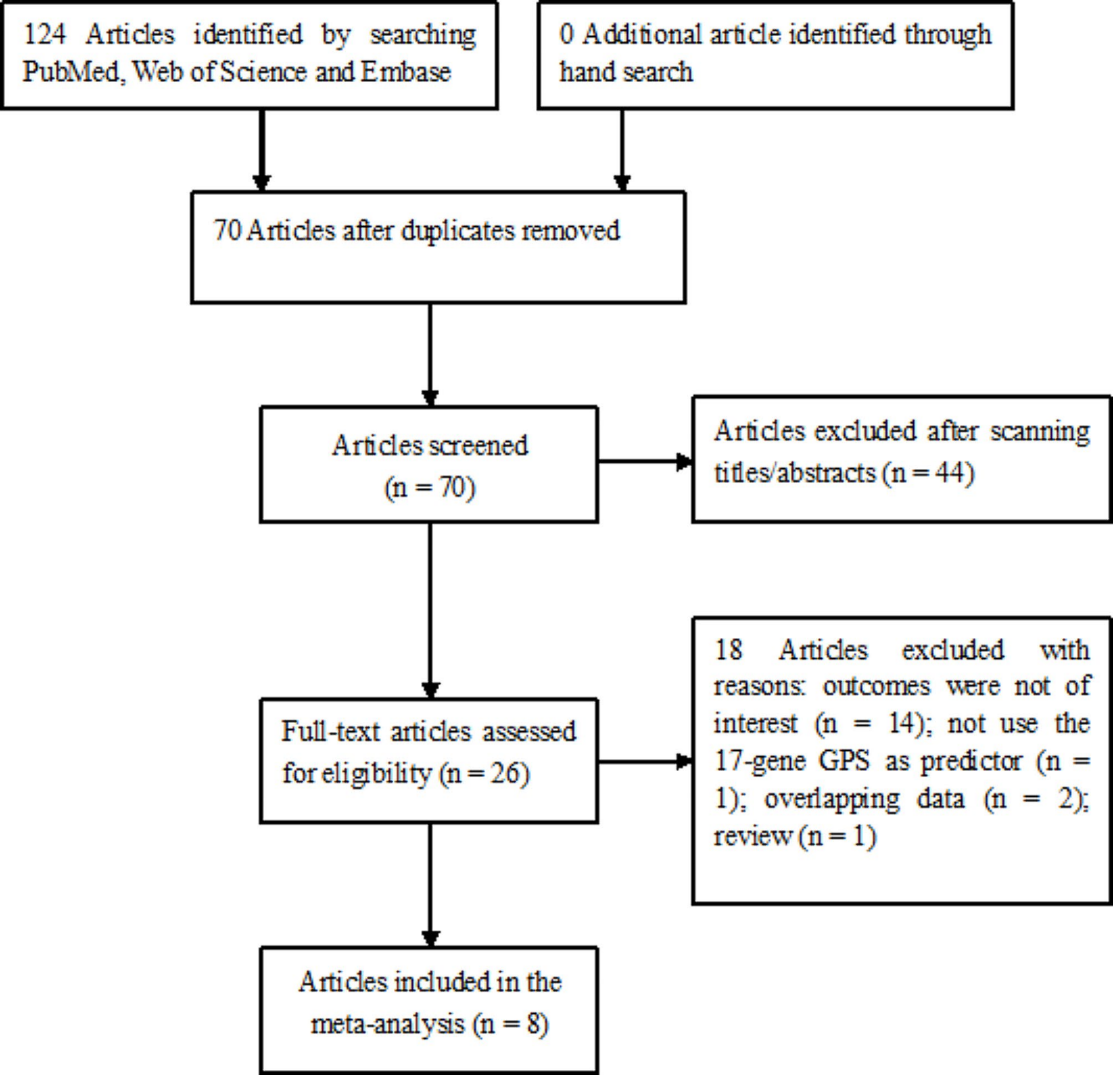
Flow chart of the study selection process

The flow chart of the study selection process is presented in Fig. 1. Briefly, the primary searching of database produced 124 potentially relevant articles. Seventy articles were reserved after removal of duplicate records. After scanning the titles or abstracts, 26 full-text articles were retrieved for eligibility assessment. Subsequently,18 articles were further excluded mainly because of three reasons: outcomes were not of interest, the 17-gene GPS was not used as a predictor, and the data overlapped. Two articles [8, 12] were from the same population and reported the different outcomes; one [11] reported the results from 2 studies. Thus, 7 cohort studies that reported on 8 articles [8–13, 18, 19] satisfied the eligibility criteria.

Table 1 describes the summary characteristics of the included articles. All of the studies were conducted the United States and were published from 2018 to 2023. Only one study [12] had a prospective design, while the rest were retrospective. These studies included a total of 1962 patients, with sample sizes ranging between 103 and 450 cases. The mean/ median age of patients at diagnosis varied from 60.7 to 65 years. The follow-up period ranged from 20 months to 15.5 years. All of the included studies focused on clinically localized prostate cancer. The summary NOS scores of the included studies ranged from 7 to 9 points (Supplemental Table S1).

**Table 1:** Main characteristics of included studies

Author/year	Study design	Sample sizes	Age (years)	Treatment	GPS cutoff	Definition of BCR	Follow-up	Outcomes HR(95% CI)	Adjusted covariates
Magi-Galluzzi 2018 [8]	Retrospective	377	61.1 ± 6.3	RP	Per 20-unit increase	Two consecutive PSA ≥ 0.2 ng/ml or receive salvage therapy	10 years	BCR 1.62 (1.14–2.31)	Multivariable adjusted analysis
Van Den Eeden 2018 [9]	Retrospective	279	61(57–65)	RP	Per 20-unit increase	Two consecutive PSA ≥ 0.2 ng/ml or receive salvage therapy	9.8 years	BCR 2.11 (1.41–3.14) DM 2.0 (1.20–3.33) PCSM 2.69 (1.50–4.82)	National Comprehensive Cancer Network risk group
Kornberg 2019 [10]	Retrospective	215	60.7 ± 6.8	RP	Per 20-unit increase	Two consecutive PSA ≥ 0.2 ng/ml	20 months	BCR 1.46 (1.00-2.14)#	Age, race/ethnicity, PSA, PSA density, Gleason grade, positive biopsy cores, clinical stage, year of diagnosis, site of GPS biopsy, biopsy timing
Cullen 2020- CPDR [11]	Retrospective	211	62(41–76)	RP	> 40 vs. ≤40; Per 20-unit increase	Two consecutive PSA ≥ 0.2 ng/ml or receive salvage therapy	5.2 years	BCR 3.22(1.67–6.22)* 3.67(2.37–5.69)*	Clinical T-Stage, PSA, Biopsy Gleason score
Cullen 2020-KPNC [11]	Retrospective	103	61(58–65)	RP	> 40 vs. ≤40	Two consecutive PSA ≥ 0.2 ng/ml or receive salvage therapy	9.8 years	BCR 7.07 (5.71–8.79) DM 5.42 (3.83–7.77) PCSM 3.43 (1.49–8.85)	Clinical T-Stage, PSA, Biopsy Gleason score
Brooks 2021 [12]	Prospective	428	61 ± 6	RP	Per 20-unit increase	—	15.5 years	DM 2.24 (1.49–3.53) PCSM 2.30 (1.45–4.36)	Preoperative PSA, clinical stage, and biopsy grade
Helfand 2022 [13]	Retrospective	141	64(57–68)	RP	> 40 vs. ≤40; Per 20-unit increase	Two consecutive PSA ≥ 0.2 ng/ml or receive salvage therapy	28 months	BCR 3.00(1.43–6.72) 2.14 (1.31–3.46)	Clinical and pathologic covariates
Canter 2023 [18]	Retrospective	450	65(60–69)	RT, ADT	Per 20-unit increase	—	61 months	DM 4.62(2.63–8.10)	National Comprehensive Cancer Network risk group
Janes 2023 [19]	Retrospective	238	64.2 ± 6.6	RT, ADT	> 40 vs. ≤40; Per 20-unit increase	—	7.6 years	DM 3.49 (1.25–12.35) 4.28 (2.43–7.75) PCSM 5.42 (1.39–36.37) 6.11 (2.93–14.33)	National Comprehensive Cancer Network risk group

Abbreviations: HR, hazard ratio; CI, confidence interval; PCa, prostate cancer; PSA, prostate-specific antigen; GPS, Genomic Prostate Score; DM, distant metastases; BCR, biochemical recurrence; PCSM, prostate cancer–specific mortality; RP, radical prostatectomy; RT, radiation therapy; ADT, androgen deprivation therapy; CPDR, Center for Prostate Disease Research; KPNC, Kaiser Permanente Northern California. #recalculated from per 5-unit increase. * Results pooled from subgroups

All the eligible were deemed high-quality based on the criteria of the NOS.

### Distant metastases

**Fig. 2:**
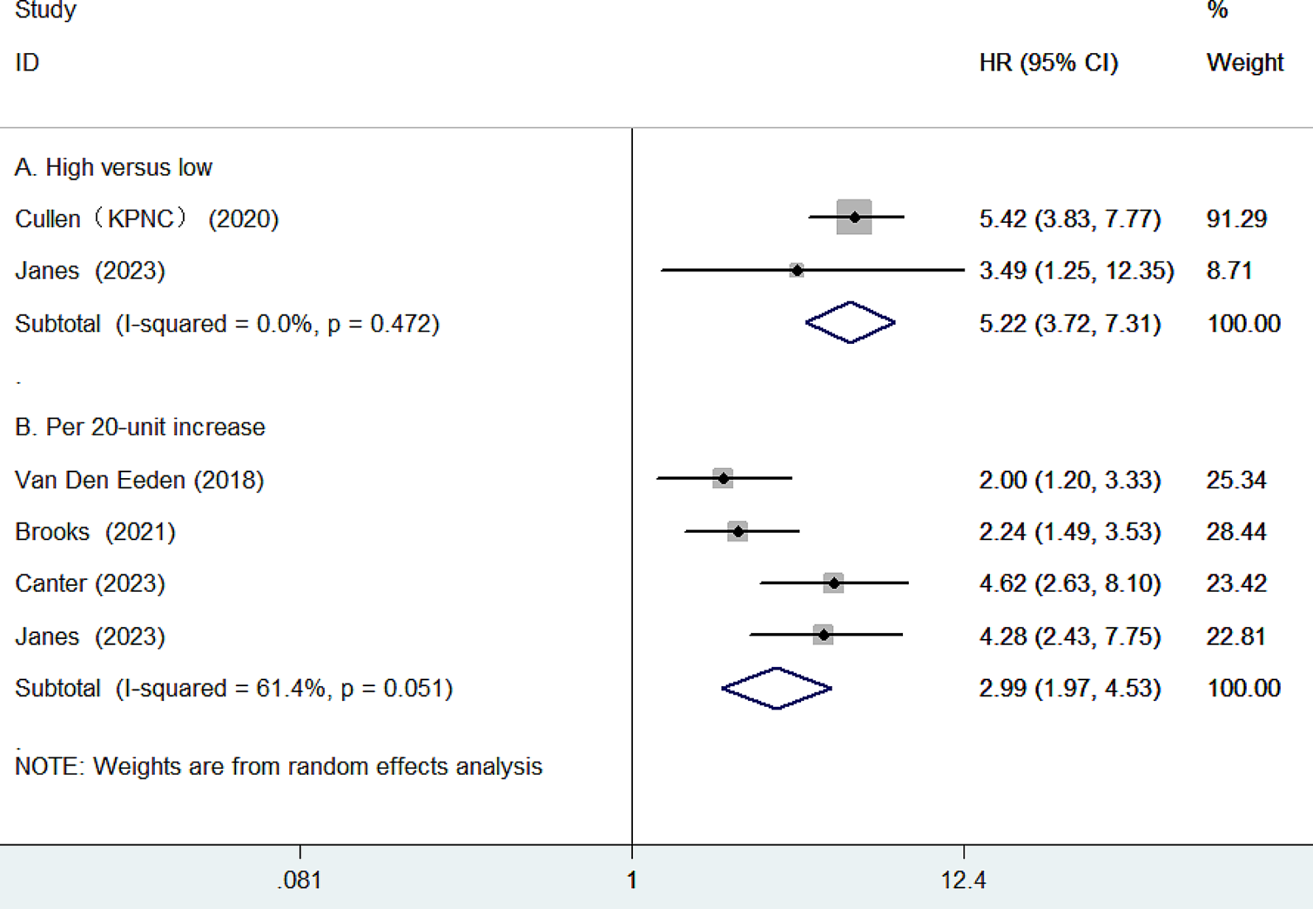
Forest plots showing the pooled HR with 95% CI of distant metastases for the high versus low Genomic Prostate Score (**A**) and per 20-unit increase in Genomic Prostate Score (**B**)

Five studies [9, 11, 12, 18, 19] reported results on the association between GPS and distant metastases. As presented in Fig. 2A, the pooled HR of distant metastases was 5.22 (95% CI 3.72–7.31) for the high versus the low GPS group. There was no evidence of significant heterogeneity (*I*^2^ = 0.0%, *p* = 0.472) between studies. Furthermore, as presented in Fig. 2B, per 20-unit increase in GPS was also significantly associated with distant metastases (HR 2.99; 95% CI 1.97–4.53), with evidence of significant heterogeneity (*I*^2^ = 61.4%, *p* = 0.051). Leave-one-out sensitivity analysis, the pooled HR ranged from 2.60 to 3.43 (All *p*-values less than 0.001) when analyzing GPS as a continuous variable. Subgroup analysis indicated that the prognostic value of per 10-point increase in GPS was stronger in patients receiving radiation therapy (HR 4.45; 95% CI 2.97–6.67) [18, 19] compared to those with receiving radical prostatectomy (HR 2.14; 95% CI 1.54–2.97) [11, 12].

### Biochemical recurrence

**Fig. 3:**
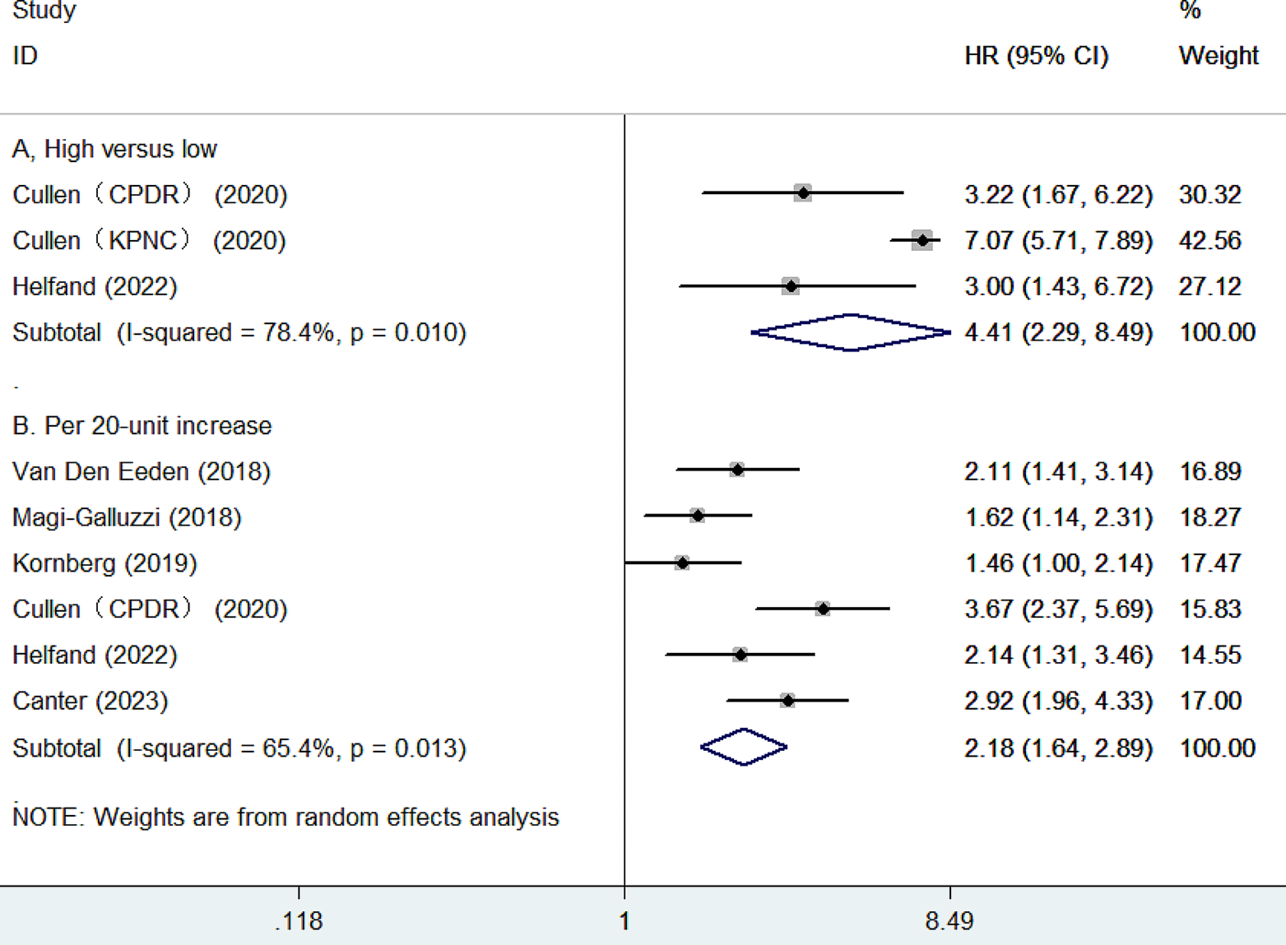
Forest plots showing the pooled HR with 95% CI of biochemical recurrence for the high versus low Genomic Prostate Score (**A**) and per 20-unit increase in Genomic Prostate Score (**B**)

Six studies [8–11, 13] reported data on the association between GPS and biochemical recurrence. As presented in Fig. 3A, the pooled HR of biochemical recurrence was 4.41 (95% CI 2.29–8.49) for the high versus the low GPS group. There was evidence of significant heterogeneity (*I*^2^ = 78.4%, *p* = 0.010) between studies. Moreover, as presented in Fig. 3B, per 20-unit increase in GPS was significantly associated with biochemical recurrence (HR 2.18; 95% CI 1.64–2.89), with evidence of significant heterogeneity (*I*^2^ = 65.4%, *p* = 0.013). Leave-one-out sensitivity analysis showed that the pooled HR ranged from 1.97 to 2.37 (All *p*-values less than 0.001) when analyzing GPS as a continuous variable. Subgroup analysis showed that the prognostic value of per 10-point increase in GPS was stronger among studies with more than 5-year follow-up (HR 2.43; 95% CI 1.70–3.46) compared to those with less than 5-year follow-up (HR 1.71; 95% CI 1.18–2.48). moreover, when the analysis restricted in studies with retrospective designs, the pooled HR of biochemical recurrence was 4.41 (95% CI 2.29–8.49). We did not observe publication bias for the association of per 20-unit increase in GPS with biochemical recurrence according to the Egger’s test (*p* = 0.265) and the Begg’s test (*p* = 0.260).

### Prostate cancer–specific mortality

**Fig. 4:**
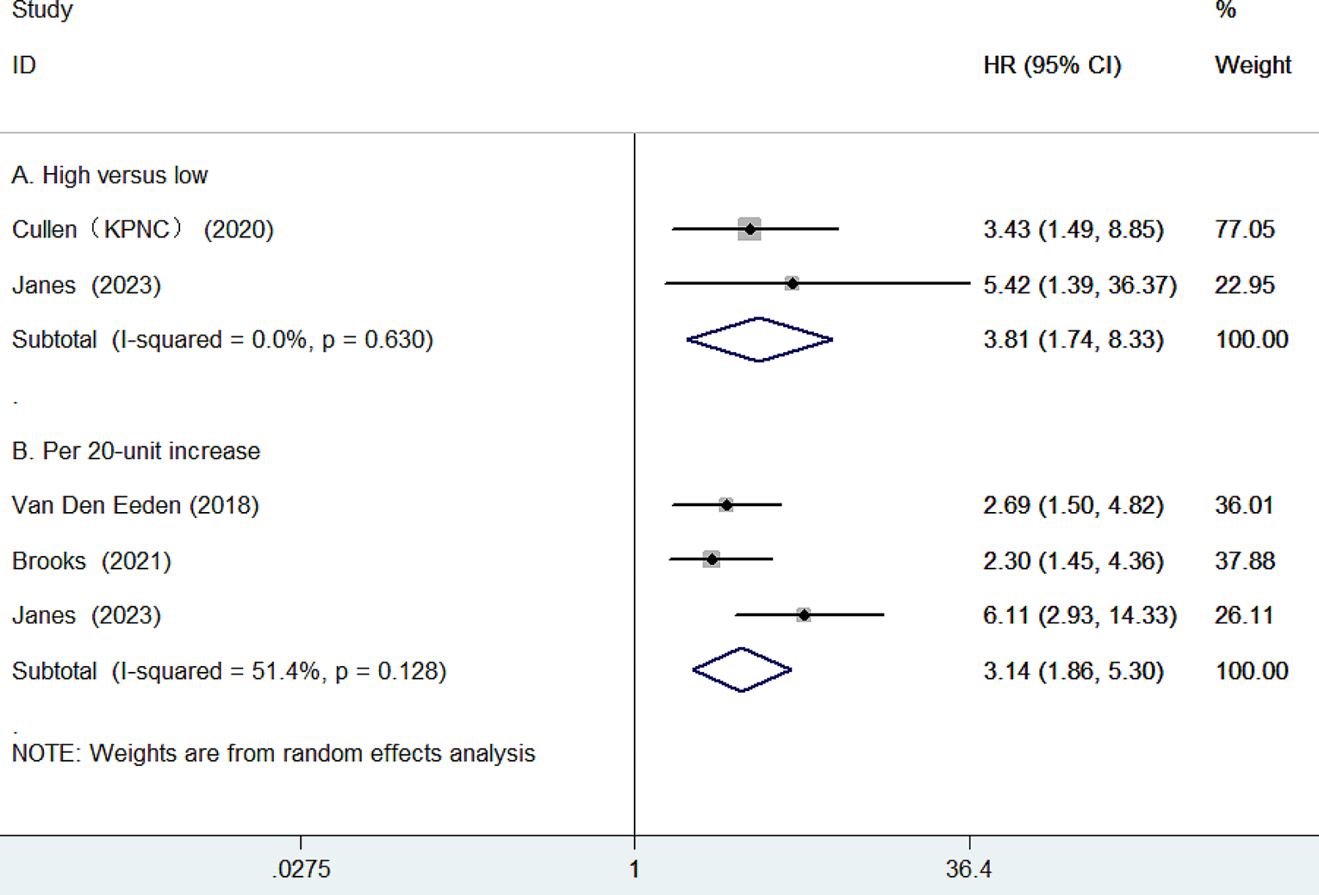
Forest plots showing the pooled HR with 95% CI of prostate cancer–specific mortality for the high versus low Genomic Prostate Score (**A**) and per 20-unit increase in Genomic Prostate Score (**B**)

Four studies [9, 11, 12, 19] reported results on the association between GPS and PCSM. As presented in Fig. 4A, the pooled HR of PCSM was 3.81 (95% CI 1.74–8.33) for the high versus the low GPS group. There was no evidence of significant heterogeneity (*I*^2^ = 0.0%, *p* = 0.630) between studies. Furthermore, as presented in Fig. 4B, per 20-unit increase in GPS was significantly associated with PCSM (HR 3.14; 95% CI 1.86–5.30), with evidence of significant heterogeneity (*I*^2^ = 51.4%, *p* = 0.128). The pooled HR was 2.48 (95% CI 1.66–3.70) when excluding Janes et al’s study [19] from the overall analysis.

## Discussion

The present meta-analysis demonstrated that a higher 17-gene GPS significantly predicted distant metastases, biochemical recurrence, and PCSM in patients with clinically localized prostate cancer. Compared to those with low GPS (≤ 40 points), prostate cancer patients with a high GPS (> 40 points) had a 5.22-fold, 4.41-fold, and 3.81-fold increased risk of distant metastases, biochemical recurrence, and PCSM, respectively. Similarly, when analyzing the GPS as a continuous variable, per 20-point increase in GPS was associated with a 2.99-fold, 2.18-fold, and 3.14-fold increased risk of distant metastases, biochemical recurrence, and PCSM, respectively. These findings suggest that 17-gene GPS can serve as a promising prognostic biomarker for predicting adverse outcomes in patients with clinically localized prostate cancer.

Our subgroup analysis by treatment option showed that the value of per 10-point increase in GPS was stronger in patients receiving radiation therapy than in those receiving radical prostatectomy. This result reveals that the 17-gene GPS potentially can help clinicians make appropriate treatment options in clinically localized prostate cancer. Moreover, the impact of per 10-point increase in GPS for predicting biochemical recurrence became stronger with prolonged follow-up duration. This finding indicates that the 17-gene GPS can be used to estimate the long-term clinical outcomes of these patients.

Adverse pathology is a surrogate indicator of prostate cancer aggressiveness. It is crucial to accurately evaluate adverse pathology in order to select the candidates for active surveillance. Recent studies have provided substantial evidence linking elevated GPS to adverse prostate cancer pathology. In men who enrolled in active surveillance but later underwent radical prostatectomy, per 5-unit increase in GPS conferred a 16% higher risk of adverse pathology [10]. For men with very low to intermediate-risk prostate cancer, a 20-unit increase in GPS led to over a threefold increased risk of adverse pathology after adjusting for other factors [20–22]. Moreover, a higher GPS also increased the probability of biopsy upgrading in prostate cancer patients undergoing active surveillance [23]. Additionally, a higher GPS was found to be an independent predictor of time to biochemical failure in men with localized prostate cancer treated with radiation therapy [18, 19]. The likelihood of biochemical recurrence in prostate cancer primarily depends on the aggressiveness of the disease. Our meta-analysis further verified that the 17-gene GPS could significantly predict biochemical recurrence in men with clinically localized prostate cancer.

The American Urological Association and American Society for Radiation Oncology has recommended the use of tissue-based multigene tests to guide treatment decisions for patients with prostate cancer [24]. One such test is the Oncotype DX GPS assay, which detects the expression of 12 genes (BGN, COL1A1, SFRP4, AZGP1, KLK2, SRD5A2, FAM13C, FLNC, GSN, GSTM2, and TPM2, TPX2) associated with prostate cancer aggressiveness, along with 5 housekeeping genes (RF1, ATP5E, CLTC, GPS1, and PGK1) [25]. This test analyzes biopsy tissue taken from prostate cancer patients. The GPS provided by the test range from 0 to 100 units, with higher values indicating a greater genomic risk of aggressive tumors.

This meta-analysis is the first to examine the prognostic value of the 17-gene GPS in patients with clinically localized prostate cancer. By pooling data from various studies, researchers can obtain a more precise estimate of the association between the 17-gene GPS and adverse outcomes in prostate cancer patients. Our analysis not only confirms the strong correlation between an elevated 17-gene GPS and negative outcomes, but also provides insight into personalized treatment strategies that may improve patient outcomes. The 17-gene GPS test quantifies genomic changes in tumor tissue, providing additional biological insight into long-term outcomes. Incorporating the 17-gene GPS into localized prostate cancer clinics may help identify patients who could benefit from active surveillance, while those with higher GPS values may benefit from immediate definitive treatment. Combining the 17-gene GPS with other prognostic factors associated with aggressiveness could improve risk stratification.

Several limitations need to be addressed in the current meta-analysis. First, most of the enrolled studies were of retrospective design, which may result in selection biases. Second, the prognostic value of GPS based on clinicopathological features of prostate cancer could not be analyzed due to a lack of available literature. Third, significant heterogeneity existed in several analyses. The likelihood for heterogeneity could be attributed to different definition in distant metastases or biochemical recurrence, age at the diagnosis, tumor stage, treatment option, and length of follow-up. Fourth, the result of publication bias for biochemical recurrence was limited by less than the recommended arbitrary minimum number of studies, and these tests for assessing publication bias are potentially unreliable [26]. Finally, it is worth noting that the focus of this meta-analysis was on clinically localized prostate cancer and not metastatic prostate cancer. Therefore, caution should be exercised when generalizing these findings to metastatic prostate cancer.

In conclusion, a higher 17-gene GPS significantly predicts the occurrence of distant metastases, biochemical recurrence, and PCSM in men with clinically localized prostate cancer. Incorporating tissue-based 17-gene GPS test into localized prostate cancer has potential to enhance risk stratification and aid in treatment decision-making. However, large-scale multicenter prospective studies are necessary to further validate these findings.

### Electronic supplementary material

Below is the link to the electronic supplementary material.

Supplementary Material 1

Supplementary Material 2

## Data Availability

Data is provided within the manuscript or supplementary information files.

## Abbreviations

GPS: Genomic Prostate Score
HR: Hazard ratios
CI: Confidence intervals
PSA: Prostate-specific antigen
PCSM: Prostate cancer–specific mortality
NOS: Newcastle-Ottawa Scale
