# Supplementary material for: Prognostic value of 17-Gene genomic prostate score in patients with clinically localized prostate cancer: a meta-analysis
Source: BMC Cancer. 2024 May 23;24:628. doi: 10.1186/s12885-024-12389-1 (PMC11112896; doi:10.1186/s12885-024-12389-1)
Supplement: Supplementary file 1 — Supplementary Material 1 [file 12885_2024_12389_MOESM1_ESM.docx]

**Supplemental Text S1 – Search strategy**

**Limits:** Publications until December 1, 2023

| 1. **PubMed Search** | Query | Items found |
| --- | --- | --- |
| #1 | Search: **(17-Gene Genomic Prostate Score) OR (Oncotype DX Prostate Cancer Assay)** | 58 |
| #2 | Search: **(((((biochemical recurrence) OR (biochemical failure)) OR (****metastasis)) OR (mortality)) OR (death)) OR (survival)** | 3,713,876 |
| #3 | Search: **(#1) AND (#2)** | **25** |

| **2. Embase Search** | Query | Items found |
| --- | --- | --- |
| #1 | Search '17-gene genomic prostate score' OR ('17 gene' AND genomic AND ('prostate'/exp OR prostate) AND ('score'/exp OR score)) | 95 |
| #2 | Search 'oncotype dx prostate cancer assay' OR (oncotype AND dx AND ('prostate'/exp OR prostate) AND ('cancer'/exp OR cancer) AND ('assay'/exp OR assay)) | 82 |
| #3 | Search #1 OR #2 | 139 |
| #4 | Search 'biochemical recurrence'/exp OR 'biochemical recurrence' OR (biochemical AND ('recurrence'/exp OR recurrence)) | 25,451 |
| #5 | Search 'biochemical failure'/exp OR 'biochemical failure' OR (biochemical AND ('failure'/exp OR failure)) | 48,718 |
| #6 | Search 'metastasis'/exp OR metastasis | 977,819 |
| #7 | Search 'mortality'/exp OR mortality | 2,012,359 |
| #8 | Search 'death'/exp OR death | 2,989,077 |
| #9 | Search 'survival'/exp OR survival | 2,290,198 |
| #10 | Search #4 OR #5 OR #6 OR #7 OR #8 OR #9 | 5,639,729 |
| #11 | Search #3 AND #10 | **66** |

|  |  |  |
| --- | --- | --- |
| **3.** **Web of Science** | Query | Items found |
| #1 | Search **17-Gene Genomic Prostate Score** (Topic) or **Oncotype DX Prostate Cancer Assay** (Topic) | 115 |
| #2 | Search **biochemical recurrence (Topic) or biochemical failure (Topic) or Metastasis (Topic) or mortality (Topic) or death (Topic) or survival (Topic)** | 5,918,703 |
| #3 | **Search #1 AND #2** | **33** |
|  |  |  |
|  |  |  |
|  |  |  |
|  |  |  |
|  |  |  |
