# Supplementary material for: Prognostic value of 17-Gene genomic prostate score in patients with clinically localized prostate cancer: a meta-analysis
Source: BMC Cancer. 2024 May 23;24:628. doi: 10.1186/s12885-024-12389-1 (PMC11112896; doi:10.1186/s12885-024-12389-1)
Supplement: Supplementary file 2 — Supplementary Material 2 [file 12885_2024_12389_MOESM2_ESM.doc]

~~Table S1 Assessment of methodological quality of the included studies~~

| Author/year | Representativeness of the exposed cohort | Selection of the non-exposed cohort | Ascertainment of exposure | Demonstration that outcome was not present at study start | Comparability of cohorts based on the design or analysis | Assessment of outcome | Enough follow-up periods (>5 years) | Adequacy of follow-up of cohorts | Total NOS score |
| --- | --- | --- | --- | --- | --- | --- | --- | --- | --- |
| Magi-Galluzzi 2018 [8] | ★ | ★ | ★ | ★ | ★ | ★ | ★ | ★ | 8 |
| Van Den Eeden 2018 [9] | ★ | ★ | ★ | ★ | ★ | ★ | ★ | ★ | 8 |
| Kornberg 2019 [10] |  | ★ | ★ | ★ | ★★ | ★ |  | ★ | 7 |
| Cullen 2020- CPDR [11] | ★ | ★ | ★ | ★ | ★★ | ★ | ★ | ★ | 9 |
| Cullen 2020-KPNC [11] | ★ | ★ | ★ | ★ | ★★ | ★ | ★ | ★ | 9 |
| Brooks 2021 [12] | ★ | ★ | ★ | ★ | ★★ | ★ | ★ | ★ | 9 |
| Helfand 2022 [13] | ★ | ★ | ★ | ★ | ★★ | ★ |  | ★ | 8 |
| Canter 2023 [18] | ★ | ★ | ★ | ★ | ★ | ★ | ★ | ★ | 8 |
| Janes 2023 [19] | ★ | ★ | ★ | ★ | ★ | ★ | ★ | ★ | 8 |

NOS, Newcastle-Ottawa Scale.
